# Ion Torrent data for the genome assembly and phylogenomic placement of mitochondrial genomes with a focus on houndsharks (Chondrichthyes: Triakidae)

**DOI:** 10.1016/j.dib.2024.110280

**Published:** 2024-03-01

**Authors:** Jessica C. Winn, Aletta E. Bester-van der Merwe, Simo N. Maduna

**Affiliations:** a Molecular Breeding and Biodiversity Group, Department of Genetics, Stellenbosch University, Stellenbosch, Western Cape, 7602, South Africa; b Department of Ecosystems in the Barents Region, Svanhovd Research Station, Norwegian Institute of Bioeconomy Research, 9925 Svanvik, Norway

**Keywords:** Next generation sequencing, Comparative mitogenomics, Taxonomy, *Mustelus*, *Triakis*

## Abstract

Here, we present, for the first time, the Ion Torrent^Ⓡ^ next-generation sequencing (NGS) data for five houndsharks (Chondrichthyes: Triakidae), which include *Galeorhinus galeus* (number of bases pairs (bp) 17,487; GenBank accession number ON652874), *Mustelus asterias* (16,708; ON652873), *Mustelus mosis* (16,755; ON075077), *Mustelus palumbes* (16,708; ON075076), and *Triakis megalopterus* (16,746; ON075075). All assembled mitogenomes encode 13 protein-coding genes (PCGs), two ribosomal (r)RNA genes, and 22 transfer (t)RNA genes (*tRNA^Leu^* and *tRNA^Ser^* are duplicated), except for *G. galeus* which contains 23 tRNA genes where *tRNA^Thr^* is duplicated. The data presented in this paper can assist other researchers in further elucidating the diversification of triakid species and the phylogenetic relationships within Carcharhiniformes (groundsharks) as mitogenomes accumulate in public repositories.

Specifications Table

**Table:** 

Subject	Biochemistry, Genetics and Molecular Biology
Specific subject area	Bioinformatics, Carcharhiniformes, Mitogenomics
Data format	Raw, Analysed
Type of data	Tables: Sequencing data (Table 1) BAM: Filtered Mitogenomic Ion Torrent^Ⓡ^ NGS data files in BAM format Data 1 - Galeorhinus_galeus_IonTorrent_Filtered_RawData.bam Data 2 - Mustelus_asterias_IonTorrent_Filtered_RawData.bam Data 3 - Mustelus_mosis_IonTorrent_Filtered_RawData.bam Data 4 - Mustelus_palumbes_IonTorrent_Filtered_RawData.bam Data 5 - Triakis_megalopterus_IonTorrent_Filtered_RawData.bam
Data collection	Genomic DNA: Standard CTAB protocol [1] or SDS-based lysis buffer (PL2) from the NucleoSpin Plant II mini kit (MACHEREY-NAGEL, Dueren, Germany); DNA quality control: Qubit 4.0 fluorometer (ThermoFisher Scientific) and LabChip^Ⓡ^ GXII Touch (PerkinElmer, Waltham, MA, USA); Library preparation: Ion Plus Fragment Library Kit (ThermoFisher Scientific) according to the manufacturer's protocol, Ion Xpress™ Plus gDNA Fragment Library Preparation User Guide (MAN0009847 K.0); NGS Sequencing: Ion GeneStudio™ S5 Prime System and postprocessing with Torrent Suite version 5.16 under default settings at the Central Analytical Facility (CAF) at Stellenbosch University.
Data source location	Fin clip tissue samples of *Galeorhinus galeus, Mustelus palumbes*, and *Triakis megalopterus* were collected along the coast of South Africa. *Mustelus asterias* and *Mustelus mosis* were sampled off the coasts of Wales and the Sultanate of Oman respectively.
Data accessibility	Repository name: Dryad Digital Repository Data identification number: NA Direct URL to data: https://doi.org/10.5061/dryad.sj3tx969h Repository name: GenBank Data identification number: ON075075, ON075076, ON075077, ON652873, and ON652874 Direct URL to data: https://www.ncbi.nlm.nih.gov/nuccore/ON075075; https://www.ncbi.nlm.nih.gov/nuccore/ON075076; https://www.ncbi.nlm.nih.gov/nuccore/ON075077; https://www.ncbi.nlm.nih.gov/nuccore/ON652873; https://www.ncbi.nlm.nih.gov/nuccore/ON652874.
Related research article	J. C. Winn, S. N. Maduna, and A. E. Bester-van Der Merwe, “A comprehensive phylogenomic study unveils evolutionary patterns and challenges in the mitochondrial genomes of Carcharhiniformes: A focus on Triakidae,” Genomics, vol. 116, no. 1, p. 110771, Jan. 2024, https://doi.org/10.1016/j.ygeno.2023.110771.

## 1 Value of the Data

Presents five newly assembled houndshark mitochondrial genomes used in the mitophylogenomic investigation in Winn et al. [2], with potential to contribute to population genomics investigations, species phylogeography delineation, and environmental DNA metabarcoding databases.

## 2 Background

Complex evolutionary patterns in the mitochondrial genome (mitogenome) of the most species-rich order, the Carcharhiniforms (groundsharks) has yielded challenges in phylogenomic reconstruction of families and genera belonging to it, particularly in the family Triakidae (houndsharks), where there are arguments for both monophyly and paraphyly. We hypothesized that opposing resolutions are a product of the *a priori* partitioning scheme selected. Accordingly, we sequenced and assembled five new mitogenomes to expand the houndshark mitogenome repository and used them in the reconstruction of the mitochondrial phylogenomic relationships within Carcharhiniforms. In the paper by Winn et al. [2], we used an extensive statistical pipeline to select a suitable partitioning scheme for inference of the mitochondrial phylogenomic relationships within Carcharhiniforms and used the multi-species coalescent model to account for the influence of gene tree discordance on species tree inference. The Ion Torrent mitogenome reads and available mitogenomes presented here can be used to further clarify the phylogenetic relationships within Triakidae as mitogenomes accumulate in public repositories.

## 3 Data Description

A total of 38,889,488 unpaired raw reads with an average of 315 bp per read were generated from the whole genome shotgun sequencing of five triakid species with the Ion GeneStudio™ S5 Prime System (Table 1). The raw sequencing data for the whole genome has been uploaded as a BioProject onto the SRA database, but it has been suppressed until release of a related manuscript. Table 1 displays the difference in length of mitogenomes assembled using the reference and ‘hybrid’ techniques, whereby the reads that mapped to the reference mitogenome were fed into a *de novo* assembly pipeline.

**Table 1:** Summary of Ion Torrent sequencing output and mitogenome assembly statistics for the five newly assembled Triakidae mitogenomes.

Species	Total # bases	# bases *Q* ≥ 20^a^	Total # reads	Mean read length (bp)	Reference assembly		‘Hybrid’ assembly	
					# reads mapped	Contig size (bp)	# of contigs	Size of largest contig (bp)
*Galeorhinus galeus*	1743,051,553	1575,927,367	5546,864	314	1152	16,758	3	16,709
*Mustelus asterias*	2198,467,513	1963,551,455	7071,002	310	3193	16,763	2	16,928
*Mustelus mosis*	2532,718,240	2303,772,449	7794,623	323	3201	16,755	1	16,883
*Mustelus palumbes*	3766,787,186	3467,446,834	11,635,770	323	4375	16,762	3	16,637
*Triakis megalopterus*	2085,956,192	1919,432,622	6841,229	304	5364	16,765	1	16,871

a Q: Phred quality score.

## 4 Experimental Design, Materials and Methods

### 4.1 Ion genestudio™ S5 data processing

For the five houndshark species for which sequencing data was generated, sequence quality was checked in FastQC and adaptors and poor-quality bases (Phred score below 20) were trimmed and reads shorter than 25 bp were removed in Torrent Suite Version 5.16. Raw reads were aligned to the *Mustelus mustelus* mitogenome (NC_039629.1; [3]) using the Geneious read mapper with medium sensitivity settings and five iterations in Geneious Prime (version 2019.1.3) [4]. The reads that mapped to the reference mitogenome were then saved in BAM format as filtered Ion Torrent reads (Data 1–5). These filtered reads were fed into a *de novo* assembly pipeline (‘hybrid’ assembly) in SPAdes v.3.15 [5] with the input set for unpaired Ion Torrent reads with 8 threads, kmers 21,33,55,77,99,127, the careful option to reduce the number of mismatches and short indels and all other parameters left as default. The resulting complete mitochondrial genomes for each houndshark species were annotated using MitoAnnotator in MitoFish v.3.85 [6,7] and deposited on GenBank. The process of filtering reads using a reference mitogenome and then mapping the filtered reads *de novo* produced a higher quality assembly, minimising reference-bias while reducing the computational demands of straight *de novo* assembly.

## Limitations

Not applicable.

## Ethics Statement

The authors have read and confirmed that this article conforms to the ethical requirements for publication in Data in Brief. We confirm that the current work does not involve human subjects, animal experiments, or any data collected from social media platforms.

## CRediT authorship contribution statement

**Jessica C. Winn:** Conceptualization, Methodology, Formal analysis, Investigation, Data curation, Writing – original draft, Writing – review & editing, Visualization. **Aletta E. Bester-van der Merwe:** Conceptualization, Resources, Writing – review & editing, Supervision, Project administration, Funding acquisition. **Simo N. Maduna:** Conceptualization, Methodology, Formal analysis, Investigation, Data curation, Writing – original draft, Writing – review & editing, Supervision.

## Data Availability

Ion Torrent data for the genome assembly and phylogenomic placement of mitochondrial genomes with a focus on houndsharks (Chondrichthyes: Triakidae) (Original data) (Dryad).
